# Dietary patterns associated with renal impairment in the Northern Ireland Cohort for the Longitudinal Study of Ageing (NICOLA)

**DOI:** 10.1007/s00394-021-02579-z

**Published:** 2021-05-07

**Authors:** Euan N. Paterson, Charlotte E. Neville, Sara M. Wallace, Jayne V. Woodside, Frank Kee, Ian S. Young, Sharon Cruise, Bernadette McGuinness, Alexander P. Maxwell, Gareth J. McKay

**Affiliations:** Centre for Public Health, Queen’s University Belfast, Belfast, Northern Ireland

**Keywords:** Dietary pattern, Nutrition, EGFR, Renal function, NICOLA

## Abstract

**Background:**

Dietary-based primary prevention guidelines for chronic kidney disease (CKD) treatment are lacking due to limited evidence. Single nutrient intake studies do not account for complex dietary interactions. We assessed associations between dietary patterns and renal function in the Northern Ireland Cohort for the Longitudinal Study of Ageing (NICOLA).

**Design:**

A cross-sectional observational study used NICOLA baseline dietary data collected between February 2014 and March 2016 via a food frequency questionnaire for 2590 participants aged ≥ 50 years. Principal component analysis identified *a posteriori* dietary patterns. Renal function was characterised by estimated glomerular filtration rate (eGFR) using serum creatinine and cystatin-C. Associations were assessed according to quintiles of dietary pattern adherence and multivariable regression analysis examined associations with eGFR.

**Results:**

Variation in three dietary patterns was significantly associated with eGFR. After adjustment for potential confounders, participants with least adherence to the ‘healthy’ dietary pattern 1 had a mean eGFR 3.4 ml/min/1.73m^2^ (95% confidence interval, [CI] − 5.0, − 1.7,* p* < 0.001) lower than the most adherent. Those with lowest adherence to the ‘unhealthy’ dietary pattern 2 had a mean eGFR 1.9 ml/min/1.73m^2^ (CI 0.2, 3.5, *p* = 0.03) higher than those with highest adherence. Participants with lowest adherence to dietary pattern 3, characterised by a high consumption of alcohol and coffee, had a mean eGFR 1.8 ml/min/1.73m^2^ (− 3.5, − 0.01, *p* = 0.05) lower than those with greatest adherence.

**Conclusions:**

Our findings identify independent associations between dietary patterns and eGFR. These findings can inform the development of diet-related primary prevention advice for CKD.

**Supplementary Information:**

The online version contains supplementary material available at 10.1007/s00394-021-02579-z.

## Introduction

Dietary contributions to the aetiology of chronic kidney disease (CKD) are not well characterised and, as a result, guidelines for primary prevention specific to CKD are lacking due to limited published evidence. Existing dietary guidelines target the primary prevention of important risk factors for CKD, including hypertension [1, 2] and type 2 diabetes, [3, 4] and the secondary prevention of hypertension, including factors related to vascular calcification, hyperkalaemia, and disease progression in CKD [5].

Diets in free-living populations are variable and complex and comprise a myriad of foods, eaten separately or together as meals. Given this complexity and the inherently multidimensional impact of foods (i.e. individual nutrient effects are co-dependent on other constituents of the diet), the association with health outcomes may depend on overall dietary pattern rather than single macro- or micronutrient intake [3]. Single nutrient intake studies do not sufficiently account for competitive, compositional and synergistic interactions between the constituent vitamins, minerals, phytonutrients, etc. found in foods, and may not confer observable benefits for free-living populations [3]. Dietary pattern approaches are now reflected in contemporary dietary recommendations for the prevention and management of conditions, such as cardiovascular disease [1, 2] and type 2 diabetes [3, 4].

Conceptually healthier interventions based on dietary patterns such as the Dietary Approaches to Stop Hypertension (DASH) diet have been shown to be associated with reduced CKD risk [6–11] and progression [12]. Likewise, traditional location-specific dietary patterns such as the Mediterranean diet, have also been associated with lower CKD risk [11, 13–20] and reduced CKD progression [21] in several populations. However, high levels of adherence to *a-priori* derived dietary patterns, such as the Mediterranean diet, can necessitate changes in food choice and food preparation methods that may present a barrier to adherence.

Studies to identify a posteriori dietary patterns in local populations and to examine the associations with renal health have been conducted in diverse populations including cohorts in the USA [6, 22, 23], Taiwan [19, 24], Brazil [18], and China [25]. A dietary pattern characterised by consumption of red meat, alcohol, sugar-sweetened beverages, and a low intake of fish, chicken, sweets, and salt was more common in those with CKD and those on haemodialysis in a Brazilian population [18]. In an American cohort (The Multi-Ethnic Study of Atherosclerosis), adherence to a dietary pattern characterised by consumption of fruits, vegetables, wholegrains, and low-fat dairy was associated with a lower urinary albumin/creatinine ratio [22]. Another study conducted in eight south-eastern US states identified a variety of local dietary patterns including a plant-based diet and a traditional “Southern” diet of meat, fried food, and sweetened beverages. Individuals with CKD and stronger adherence to the Southern dietary pattern had higher mortality rates compared to those with CKD consuming a plant-based dietary pattern [23].

In a Taiwanese population, a dietary pattern characterised primarily by a greater consumption of meat, offal, processed food, rice and flour products,, and low consumption of dark-coloured fruit and vegetables, was associated with a small, increased risk of poorer renal function [19]. In a Taiwanese population with diabetes, three dietary patterns were identified; a high-fat diet, a traditional Chinese diet, and a diet rich in fish and vegetables. The dietary pattern rich in fish and vegetables was associated with better renal function compared to the others [24]. In contrast, a Chinese population-based study of a posteriori dietary patterns in an area where industrial pollutants are commonly reported to contaminate fresh food products, identified an association between a traditional Chinese diet, resembling the Mediterranean diet, and increased risk of CKD [25].

These studies highlight the benefits of evaluating the effects of conceptually healthy and naturally existing dietary patterns in a region-specific manner. However, there is little evidence in British and Irish populations, which likely differ markedly in their dietary habits and the type and quality of available foods compared to other populations. In the Irish Nun Eye Study (INES [26]), greater adherence to an unhealthy dietary pattern was associated with a reduction in renal function equivalent to 14 years of the mean annual age-related renal decline as reported in the Cardiovascular Health Study [27]. However, the INES comprised cloistered nuns and generalisability to the wider population cannot be assumed. Therefore, this study aimed to identify associations between culturally relevant dietary patterns and renal function in a population of older adults from the Northern Ireland Cohort for the Longitudinal Study of Ageing (NICOLA). The findings will help to inform diet-related primary prevention advice for CKD in older people.

## Methods

Data from Wave 1 of NICOLA were used to test associations between renal function and dietary patterns specific to the Northern Ireland population. This study collected health and lifestyle data from 8452 free-living individuals in Northern Ireland, aged 50 years and over with planned 10-year follow-up assessments for consenting participants. Baseline data were collected between December 2013 and March 2016 (NCT number: NCT01938898 [28]). The study adhered to principles outlined in the Declaration of Helsinki.

Data collection included a health assessment, a self-completion questionnaire and a computer-assisted personal interview. Blood samples were collected as part of the health assessment. Renal function was assessed as the estimated glomerular filtration rate (eGFR) calculated using the combined CKD-EPI equation for serum creatinine and cystatin-C from a blood sample collected at a single time point [5]. Dietary intake was assessed using the validated 130-item food frequency questionnaire (FFQ), previously used by the European Prospective Investigation of Cancer (EPIC) study [29]. For each food item on the FFQ, participants specified their frequency of consumption (in the previous 12 months) ranging from never or less than once per month, 1–3 times per month, once a week, 2–4 per week, 5–6 per week, once a day, 2–3 per day, 4–5 per day and 6 + times per day which were converted into estimated daily gram weights using standardised Food Portion Sizes [30].

### Other variables

Systolic blood pressure (SBP) and diastolic blood pressure (DBP) measurements were the average of two independent readings. Diabetic status was defined using a combination of participant percentage haemoglobin A1c (HbA1c > 6.5%), diabetic medication use or self-reported diabetes. Self-reported medication use was defined according to the Anatomical Therapeutic Chemical (ATC) Classification System based on the active ingredients of drugs according to the organ or system on which they act and their therapeutic, pharmacological and chemical properties. Educational attainment was classified as completing primary, secondary or tertiary level education. Smoking status was characterised as current, past or non-smoker. Alcohol consumption was categorised into three groups: non-drinker, drinker and ex drinker.

### Statistical methods

Statistical analyses were conducted using SPSS v24 (IBM, Chicago, Illinois, USA). Continuous variables were summarised using means and standard deviations. Categorical variables were summarised using frequencies and percentages. The dietary pattern analysis has been described previously [29]. In brief, individual foods and drinks from the FFQ were clustered into 34 food groups based on food type. These food groups were used to generate dietary patterns using principal component analysis (PCA) with orthogonal (varimax) rotation. The PCA generated factor loadings for each food group. The number of factors retained was based on the number of points above the breakpoint on the scree plot. Food groups with factor loadings greater than 0.2 were retained as they were considered informative in describing the dietary pattern. Dietary pattern adherence scores were computed for each participant by summing the intakes of food groups weighted by their factor loading. Dietary pattern scores were categorised into quintiles of adherence to each dietary pattern. The highest quintile, reflecting greatest conformity to the pattern, was designated as the reference category for regression analyses.

Differences in mean eGFR between quintiles of dietary pattern adherence were assessed using one-way analysis of variance (ANOVA). Adjustment for multiple comparison was carried out using Student–Neuman–Keuls (SNK) *post hoc* test. Differences in categorical variables between quintiles of adherence were assessed using the Kruskal–Wallis test with *post hoc* pairwise Mann–Whitney U tests to determine the location of differences. Multivariable linear regression analysis was used to assess associations between eGFR and dietary pattern adherence. Adjusted models controlled for age, sex, diabetes status, SBP, smoking status, alcohol consumption, waist circumference, high-density lipoprotein (HDL), low-density lipoprotein (LDL), lipid-modifying agent use, antihypertensive and diabetes drug use, multiple-deprivation score, and education level. In a sensitivity analysis, we tested for associations of interactions between eGFR and dietary pattern adherence by BMI category and for those 65 < age ≤ 65 years. For all analyses,* p *< 0.05 was considered statistically significant. Study datasets are available from the corresponding author following reasonable request.

## Results

NICOLA baseline characteristics for the 2590 participants with both dietary pattern and renal function data are shown in Table 1. The sample included 1224 (47%) males and 1366 (53%) females with a mean age of 64.4 years (SD = 9.3). Mean systolic/diastolic blood pressure was 133/81 mmHg (SD = 18/11), and mean waist circumference was 95.1 cm (SD = 13.9), which is above the healthy central adiposity threshold for both men and women. The mean BMI for all participants was 28.7 kg/m^2^ with 589 (23%) having a BMI < 25 kg/m^2^, 1139 (44%) BMI ≥ 25 kg/m^2^ but < 30 kg/m^2^ and 862 (23%) had a BMI ≥ 30 kg/m^2^. Mean HDL cholesterol was 1.61 mmol/L (SD = 0.44) and mean LDL cholesterol was 3.34 mmol/L (SD = 1.10). Nine percent of participants had diabetes mellitus and 40% were hypertensive (defined as a SBP ≥ 140 mm Hg or DBP ≥ 90 mm Hg or if use of antihypertensive medication was self-reported). The mean eGFR was 73.5 ml/min/1.73m^2^ (SD = 16.3). The majority (85%) of participants had completed at least secondary level education, 54% had never smoked, and 69% currently consumed alcohol. A lipid-modifying agent was being used by 34% of participants; 6% used medication to treat their diabetes, and 2% used antihypertensive medication.

**Table 1:** Population characteristics

Variable	Mean (SD)
Age (years)	64.4 (9.3)
Male, * n *(%)	1224 (47.3)
Body fat (%)	43.8 (6.4)
Waist circumference (cm)	95.1 (13.9)
Mean BMI (kg/m^2^)	28.7 (5.1)
BMI < 25 kg/m^2^	589 (23)
25 ≥ BMI < 30 kg/m^2^	1139 (44)
BMI ≥ 30 kg/m^2^	862 (23)
Systolic blood pressure (mmHg)	133 (18)
Diastolic blood pressure (mmHg)	81 (11)
LDL (mmol/L)	3.34 (1.10)
HDL (mmol/L)	1.61 (0.44)
eGFR CKD-EPI SCr SCys (ml/min/1.73m^2^)	73.5 (16.3)
CKD stages 1–2	2082 (82)
CKD stage 3	350 (14)
CKD stage 4–5	117 (4)
Diabetes mellitus, * n * (%)	219 (8.5)
Hypertensive, * n *(%)	1038 (40)
Using lipid modifying agents, * n * (%)	869 (34)
Using antihypertensive drugs, * n * (%)	59 (2.3)
Using drugs for diabetes control, * n *(%)	149 (5.8)
Education level:	
Primary or less, * n * (%)	375 (15)
Secondary, * n *(%)	1125 (44)
Tertiary, * n * (%)	1089 (41)
Smoking	
Never, * n * (%)	1389 (54)
Ex, * n * (%)	966 (37)
Current, * n * (%)	234 (9)
Alcohol consumption	
Current, * n * (%)	1785 (69)
Ex, * n * (%)	374 (14)
Never, * n * (%)	430 (17)

Hypertension was defined as a systolic blood pressure ≥ 140 mm Hg or diastolic blood pressure ≥ 90 mm Hg or if use of antihypertensive medication was self-reported. Medication use was defined according to the Anatomical Therapeutic Chemical (ATC) Classification System that classifies the active ingredients of drugs according to the organ or system on which they act and their therapeutic, pharmacological and chemical properties

*SD* Standard deviation, *BMI* body mass index, *LDL* Low-density lipoprotein cholesterol, *HDL* High-density lipoprotein cholesterol, *eGFR* estimated glomerular filtration rate (CKD-EPI SCr and SCys), C*KD* chronic kidney disease

Three dietary patterns met the retention criteria of Eigenvalues greater than 1 and above the scree plot breakpoint. The food components and factor loadings of each dietary pattern are described in Table 2. A healthy dietary pattern 1 was defined by high factor loadings for (and more frequent consumption of) vegetables, wholegrains, fruit, oily fish, dairy, and white fish. An unhealthy dietary pattern 2 was characterised by high factor loadings for (and greater intake of) processed meat, white bread, confectionary, fried potatoes, red meat, and added sugar. Dietary pattern 3 was characterised by high factor loadings for (and greater intake of) coffee, wine, beer, other alcohol, refined grains, crisps, and lasagne/pizza but negative factor loadings for (and low intake of) butter, margarine, confectionary, higher fibre breads, cereals, natural potatoes, and tea. Together, these three dietary patterns accounted for approximately 22.3% of the total variance in this population.

**Table 2:** Dietary patterns and factor loadings identified using principal components analysis

Dietary pattern 1 Total variance: 9.54%		Dietary pattern 2 Total variance: 6.82%		Dietary pattern 3 Total variance: 5.90%	
Food group	Factor loading	Food group	Factor loading	Food group	Factor loading
Vegetables	0.698	Processed meat	0.578	Coffee	0.455
Wholegrains	0.561	White bread	0.516	Wine	0.436
Fruit	0.554	Confectionary	0.507	Beer	0.415
Oily fish	0.478	Fried potatoes	0.492	Other alcohol	0.402
Dairy e.g. cream, yogurt, cheese	0.474	Red meat	0.474	Refined grains	0.272
White fish	0.471	Added sugar i.e. sugar added to tea, coffee, cereal	0.379	Crisps	0.252
Soups, sauces, condiments	0.450	Other soft drinks	0.376	Lasagne/pizza	0.251
Refined grains	0.424	Crisps	0.359	Butter	− 0.210
Higher fibre breads	0.377	Butter	0.313	Margarine	− 0.266
Poultry	0.349	Soups, sauces, condiments	0.313	Confectionary	− 0.268
Eggs	0.323	Lasagne/pizza	0.291	Higher fibre breads	− 0.357
Nuts	0.304	Margarine	0.278	Cereals	− 0.384
Tofu	0.233	Natural potatoes	0.265	Natural potatoes	− 0.425
Lasagne / pizza	0.231	Other alcohol	0.212	Tea	− 0.530
Cereals	0.202				

The difference in mean renal function for participants, divided by quintiles of adherence to dietary patterns is shown in Table 3. For the healthy dietary pattern 1, those in the lowest three quintiles of adherence had significantly poorer renal function (eGFR) than those with the highest intake of foods associated with this dietary pattern: least adherent: eGFR 71.3 ml/min/1.73m^2^ (SD: 16.6; *p *< 0.001) compared to the highest adherence eGFR 76.0 ml/min/1.73m^2^ (15.6; reference category). These differences remained significant in the adjusted model where the least adherent quintile had an eGFR mean difference of − 3.4 ml/min/1.73m^2^ (95% confidence interval − 5.0, − 1.7; *p* < 0.001) compared to the most adherent reference category. The association between adherence to dietary pattern 1 and eGFR was significant in both the unadjusted and adjusted models (p for trend < 0.001). In a sensitivity analysis dichotomised by 65 < age ≥ 65 years, the effect size was diminished in those aged ≥ 65 years but was no longer significant in the smaller sample size, although the trend remained the same (*P* = 0.09; supplementary Tables 1, 2 and 3).

**Table 3:** Mean renal function (unadjusted) and mean difference in renal function (unadjusted and adjusted) by quintiles of dietary pattern adherence

	Least adherent	*p*	Low adherence	*p*	Middle adherence	*p*	High adherence	*p*	Highest adherence	*P* for trend
	*n* = 510		*n* = 508		*n* = 520		*n* = 523		*n* = 529	
Mean eGFR (SD) ml/min/1.73m^2^	71.3 (16.6)		71.6 (16.7)		73.5 (16.0)		75.1 (16.1)		76.0 (15.3)	
Mean difference in eGFR (ml/min/1.73m^2^) from reference category (95% CI):										
Unadjusted	− 4.7 (− 6.7, − 2.8)	< 0.001	− 4.4 (− 6.4, − 2.4)	< 0.001	− 2.5 (− 4.5, − 0.6)	0.01	− 0.9 (− 2.9, 1.1)	0.37	Ref	< 0.001
Adjusted	− 3.4 (− 5.0, − 1.7)	< 0.001	− 2.7 (− 4.3, − 1.1)	0.001	− 2.5 (− 4.1, − 0.9)	0.002	− 0.9 (− 2.5, 0.7)	0.28	Ref	< 0.001
Dietary pattern 2	*n* = 517		*n* = 512		*n* = 521		*n* = 519		*n* = 521	
Mean eGFR (SD) ml/min/1.73m^2^	76.2 (16.2)		72.7 (16.4)		72.3 (16.2)		72.6 (16.3)		73.8 (16.3)	
Mean difference in eGFR (ml/min/1.73m^2^) from reference category (95% CI):										
Unadjusted	2.3 (0.4, 4.3)	0.02	− 1.2 (− 3.1, 0.8)	0.25	− 1.6 (− 3.5, 0.4)	0.12	− 1.2 (− 3.2, 0.8)	0.24	Ref	0.04
Adjusted	1.9 (0.2, 3.5)	0.03	− 0.3 (− 1.9, 1.3)	0.71	− 0.7 (− 2.3, 0.9)	0.38	− 1.2 (− 2.8, 0.4)	0.15	Ref	0.02
Dietary pattern 3	*n* = 514		*n* = 526		*n* = 521		*n* = 511		*n* = 518	
Mean eGFR (SD) ml/min/1.73m^2^	70.0 (15.9)		70.4 (16.5)		73.9 (16.5)		75.3 (15.7)		78.1 (15.3)	
Mean difference in eGFR (ml/min/1.73m^2^) from reference category (95% CI):										
Unadjusted	− 8.0 (− 10.0, − 6.1)	< 0.001	− 7.7 (− 9.6, − 5.8)	< 0.001	− 4.2 (− 6.1, − 2.2)	< 0.001	− 2.8 (− 4.8, − 0.8)	0.005	Ref	< 0.001
Adjusted	− 1.8 (− 3.5, − 0.01)	0.05	− 3.2 (− 4.8, − 1.5)	< 0.001	− 1.5 (− 3.1, 0.2)	0.08	− 0.4 (− 2.0, 1.3)	0.65	Ref	0.001

*eGFR* estimated glomerular filtration rate (CKD-EPI SCr and SCys), *CI* Confidence interval. Mean eGFR values are unadjusted. Adjusted models included age, sex, diabetes status, systolic blood pressure, smoking status, alcohol consumption, waist circumference, high-density lipoprotein, low-density lipoprotein, lipid-modifying agent use, antihypertensive and diabetes drug use, multiple-deprivation score, and education level

For the unhealthy dietary pattern 2, those in the lowest quintile of adherence had significantly better renal function than those in the reference category, i.e. the highest intake of foods associated with dietary pattern 2. Least adherent quintile: eGFR 76.2 ml/min/1.73m^2^ (16.2; *p* = 0.02) compared to the most adherent eGFR 73.8 ml/min/1.73m^2^ (16.3; reference category). These differences remained significant in the adjusted model where the least adherent quintile had an eGFR mean difference 1.9 ml/min/1.73m^2^ (0.2, 3.5], *p* = 0.03) compared to the most adherent reference category. The association between adherence to dietary pattern 2 and eGFR was significant for both the unadjusted (*p* = 0.04) and adjusted models (*p *for trend = 0.02) and this effect was largely driven by those aged ≥ 65 years (*P* = 0.006; Supplementary tables 1, 2 and 3).

For dietary pattern 3, characterised by a high consumption of alcohol and coffee, those with lower adherence had significantly poorer renal function than those with the highest intake of foods associated with this dietary pattern (least adherent quintile: eGFR 70.0 ml/min/1.73m^2^ (15.9; *p* < 0.001) compared to the most adherent quintile: eGFR 78.1 ml/min/1.73m^2^ (15.3; reference category). These differences remained significant in the adjusted model where the least adherent quintile: eGFR − 1.8 ml/min/1.73m^2^ (− 3.5, − 0.01; *p* = 0.05) compared to the most adherent reference category. The association between adherence to dietary pattern 3 and eGFR was significant for both the unadjusted (*p* for trend < 0.001) and adjusted models (p for trend = 0.001). In a sensitivity analyses stratified by those aged < 65 years remained significant (*P* = 0.002), although the association with those aged ≥ 65 years was just above the significance threshold for (*P* = 0.06; Supplementary tables 1, 2 and 3).

Sensitivity analyses by BMI categories identified no significant interaction or deviation in the associations reported between eGFR and dietary pattern adherence (data not shown).

## Discussion

In this free-living, population-based cohort, comprising adults  ≥ 50 years old in Northern Ireland, three a posteriori dietary patterns were identified. Low adherence to a dietary pattern characterised by consumption of processed meats, white bread, confectionary, fried potatoes, red meat, added sugar, etc. (dietary pattern 2), was associated with higher eGFR and this effect was stronger in those aged < 65 years. In contrast, poor adherence to the two other dietary patterns were associated with lower eGFR. These were dietary pattern 1, characterised by consumption of vegetables, wholegrains, fruits, oily fish, dairy, white fish etc., and dietary pattern 3, characterised by high intakes of drinks such as coffee, wine, beer and other alcohol, but low intakes of butter, margarine, confectionary, higher fibre breads, cereals, natural potatoes and tea. These associations were independent of diabetes status, blood pressure and other measured potential confounding factors.

There was considerable overlap between the characteristics of the dietary patterns observed and the components of other conceptually healthy and unhealthy dietary patterns. The healthy dietary pattern 1 was characterised by consumption of foods similar to those found in the Mediterranean and DASH diets, while the unhealthy dietary pattern 2 was principally comprised of foods similar to those found in “Western diets” which have been associated with conditions such as diabetes and cardiovascular disease. Likewise, both of these dietary patterns were similar to those reported previously in the geographically overlapping INES [26], wherein the observed diets were labelled as the “unhealthy” and “healthy” dietary patterns. In the INES, the unhealthy pattern was associated with significantly poorer renal function, while no associations were detected between adherence to the healthy dietary pattern and kidney function. The INES provided evidence consistent with a detrimental effect of consumption of unhealthy food types but failed to support the beneficial effects for the consumption of what would be regarded as healthy foods. In contrast, the present study, conducted in a more generalizable and free-living, but geographically similar population, confirmed the association between consumption of a naturally occurring diet similar to those in the INES unhealthy dietary pattern but also showed a protective effect of a diet rich in fruits, vegetables, and fish. Of note, the NICOLA was more than twice the size of the INES, included both men and women and as such, had greater power to detect associations with improved sensitivity of the effect sizes observed. The effect size of the association for each dietary pattern was smaller than the effect size of the association observed between the unhealthy dietary pattern and renal function in the INES. Lowest adherence to dietary pattern 2 was associated with a 1.9 ml/min/1.73m^2^ increase in eGFR compared to a 6 ml/min/1.73m^2^ increase in renal function for those in the least adherent group for the unhealthy dietary pattern in the INES. The reductions in eGFR associated with low adherence to dietary patterns 1 and 3 were closer in magnitude at 3.4 ml/min/1.73m^2^ and 1.75 ml/min/1.73m^2^. The magnitude of these associations is equivalent to a year or more of annual age-related loss of renal function as reported in the Cardiovascular Health Study [27]. Although both studies undertook age-adjusted analyses, the smaller effect size observed between dietary pattern 2 adherence and eGFR in the present study, compared to the similarly unhealthy dietary pattern observed in the INES population, may be explained by the older mean age of participants in the INES and the associated age-related decline in eGFR. Furthermore, the sensitivity analyses stratified by age in the current study suggest an age-related effect which is perhaps unsurprising, given the gradual loss of renal function as we grow older, with a mean eGFR = 66 ml/min/1.73m^2^ in those aged ≥ 65 years versus 81 ml/min/1.73m^2^ in those aged < 65 years. Given the possibility of over adjustment for age (it was included as a potential confounder in the adjusted regression models, it was used to dichotomise the study sample (65 < age ≥ 65 years) thereby reducing statistical power, and given its role in estimating glomerular filtration rate, the findings from the sensitivity analyses should be interpreted with caution.

Dietary pattern 3 from the NICOLA study did not resemble any of the dietary patterns from the INES, despite the similar geographic location. This dietary pattern was characterised by a high intake of coffee, alcohol, crisps and pizza and a low intake of high-fibre breads, spreads and tea. The non-existence of this dietary pattern in the INES population may be unsurprising among those practicing a cloistered and religious lifestyle. Although conceptually unhealthy, low adherence to this dietary pattern was associated with poorer renal function in the current study. However, this finding may yet be related to confounding influences which were not controlled for, or, may result from the high intake of coffee [31] and alcohol [32–35], or greater total fluid intake [36, 37], which have been associated with better renal function, despite the associations between alcohol and other related conditions.

Several other active food constituents in the dietary patterns observed have been previously associated with renal function. Reduced endogenous antioxidant production has been reported in individuals with end-stage renal disease (ESRD) requiring haemodialysis [38], and antioxidant supplementation has been reported to reduce the rate of decline in renal function [39]. Interestingly, a wide variety of fruits and vegetables contain substances with antioxidant capacity including vitamins A, C and E, and poly-phenolics [40, 41] and associations between xanthophyll serum carotenoid and eGFR have been previously reported [42]. Similarly, fruits and vegetables contain a wide variety of anti-inflammatory substances which regulate nitric oxide production, the cyclooxygenase pathway, prostaglandin production and macrophage function such as poly-phenolics, triterpenoids, saponins, lectins, etc. [41]. The fibre found within fruits, vegetables and grains may also be beneficial, with every 5 g increase in daily fibre intake associated with an 11% reduction in CKD incidence [43].

Oily fish are rich in long-chain, n-3 polyunsaturated fatty acids which act on the cyclooxygenase pathway and arachidonic acid pathway to reduce the production of a variety of inflammatory mediators [44]. Moreover, animal models of fish derived dietary proteins were shown to have lower urine cystatin-C concentration compared to those from casein [45]. In contrast, meat intake has been associated with increased ESRD incidence [46] and in the Singapore Chinese Health Study, where red meat intake was considered separately from other protein sources, red meat, but not fish, poultry, eggs, or dairy intake, was associated with greater incidence of ESRD [47]. Similar results were reported in the Atherosclerosis Risk in Communities (ARIC) study, where processed meats were considered as an additional, separate category and both red and processed meats were associated with incident ESRD [48]. The monosaccharide sugar fructose, typically found in high concentrations in fruits, has been implicated in renal damage. Indeed, dehydration-induced endogenous fructose production has been found to increase renal inflammation and fibrosis in animal models [49], and low fructose diets in individuals with CKD have been reported to reduce blood pressure and inflammation [50]. Such findings provide mechanistic plausibility for empirically observed associations between dietary patterns and renal health.

The findings of this study add further support to previous studies reporting associations between conceptually healthy diets and CKD risk and provide novel evidence for the association between dietary patterns and eGFR in British and Irish populations, for which there is limited data, independent of the effect of diet on diabetes risk, blood pressure and other potential confounding factors.

These analyses had several strengths. A large, population-based cohort enabled the generalisation of the findings, and unlike a number of previous studies which were limited in their ability to adjust for important confounding factors, the current study included a wide range of biological and lifestyle covariates. Dietary patterns and CKD prevalence vary by region [51, 52], and we have previously reported significant associations [26] between dietary patterns and renal function in a local population, using a more representative generalizable sample. The primary outcome variable was quantified using the CKD-EPI equation for both SCr and SCys, this equation is less affected by variations in muscle mass and diet and better predicts mortality than GFR estimated on SCr alone [53]. Examining dietary patterns instead of individual nutrients provides additional benefits for public health practice considering the interaction between dietary factors [54, 55], and the conceptually simpler public messaging for a healthy diet, although conversely fails to account for the effects of individual nutrients of interest. A posteriori dietary patterns derived specifically from a study population may also provide necessary context and relevance to regional cultures, increasing the applicability of the findings locally. Furthermore, a posteriori approaches used to identify the dietary patterns are not constrained by predetermined hypotheses that link nutrients with potential health outcomes.

The study design has several limitations. NICOLA has an inherent sample bias resulting from the recruitment of individuals aged 50 years and older. In addition, the sample excluded institutionalised individuals and those with dementia. Therefore, the generalisability of the study results may not extend to younger individuals, who may commonly adhere to other dietary patterns not identified in the current study, or to individuals with dementia in whom dietary habits may be profoundly affected. Longitudinal and experimental study designs could provide evidence of cause and effect but, at the time of this analysis, only cross-sectional data were available and so firm causal inferences cannot be drawn. Furthermore, dietary data were only captured at one time-point which may not reflect changes in dietary intakes. Direct measurement of glomerular filtration rate would provide an assessment of renal function with smaller inherent error than eGFR measures, however, eGFR was estimated using the clinically relevant CKD-EPI estimating equation and made use of the more accurate SCys and SCr-based measures which also better predicts complications associated with CKD [53, 56]. Moreover, renal function was estimated at a single time point. Although common practice in epidemiological studies, at an individual level this cannot account for diurnal and acute variations in renal function in the same way that the clinical practice of using two measures of eGFR taken at least three months apart can [5]. However, the large sample size minimises the probability of false null findings.

In summary, the findings of this study provide novel evidence for associations between culturally existing eating patterns and renal function, relevant to British and Irish populations, independent of the effect of diet on diabetes risk, blood pressure and other potential confounding factors. The results of this study will support diet-related primary prevention advice for CKD and inform future longitudinal analyses within the NICOLA.

## Supplementary Information

Below is the link to the electronic supplementary material.

Supplementary file1 (DOCX 23 kb)

## Data Availability

The data that support the findings of this study are available from the Northern Ireland Cohort for the Longitudinal Study of Ageing but restrictions apply to the availability of this data, which was used under license for the current study, and so is not publicly available. Data may, however, be made available from the corresponding authors upon reasonable request and provided there is permission from NICOLA.
