# Supplementary material for: Dietary patterns associated with renal impairment in the Northern Ireland Cohort for the Longitudinal Study of Ageing (NICOLA)
Source: Eur J Nutr. 2021 May 7;60(7):4045–54. doi: 10.1007/s00394-021-02579-z (PMC8437851; doi:10.1007/s00394-021-02579-z)
Supplement: Supplementary file 1 — Supplementary file1 (DOCX 23 kb) [file 394_2021_2579_MOESM1_ESM.docx]

**Supplementary Table 1.** Mean renal function (unadjusted) and mean difference in renal function (unadjusted and adjusted) by quintiles of dietary pattern adherence for age < 65 years.

|  | **Least**  **adherent** | **p** | **Low**  **adherence** | **p** | **Middle adherence** | **p** | **High adherence** | **p** | **Highest adherence** | **P for trend** |
| --- | --- | --- | --- | --- | --- | --- | --- | --- | --- | --- |
| **Dietary Pattern 1** | n=270 |  | n=241 |  | n=279 |  | n=278 |  | n=277 |  |
| Mean eGFR (SD) ml/min/1.73m^2^ | 77.6 (14.9) |  | 79.7 (13.1) |  | 79.9 (14.0) |  | 82.4 (12.8) |  | 82.9 (13.9) |  |
| Mean difference in eGFR (ml/min/1.73m^2^) from reference category (95% CI): | | | | | | | | | |  |
| unadjusted | -5.3 (-7.6, -3.0) | <0.001 | -3.3 (-5.6, -0.9) | 0.007 | -3.0 (-5.3, -0.7) | 0.007 | -0.5 (-2.8, 1.8) | 0.68 | Ref. | <0.001 |
| adjusted | -4.6 (-6.8, -2.3) | <0.001 | -3.1 (-5.3, -0.9) | 0.006 | -3.0 (-5.2, -0.9) | 0.005 | -0.3 (-2.4, 1.8) | 0.78 | Ref. | <0.001 |
|  | | | | | | | | | |  |
| **Dietary Pattern 2** | n=265 |  | n=239 |  | n=253 |  | n=297 |  | n=291 |  |
| Mean eGFR (SD) ml/min/1.73m^2^ | 81.7 (15.1) |  | 81.2 (13.3) |  | 80.6 (12.9) |  | 79.1 (14.0) |  | 80.5 (13.8) |  |
| Mean difference in eGFR (ml/min/1.73m^2^) from reference category (95% CI): | | | | | | | | | |  |
| unadjusted | 1.1 (-1.2, 3.4) | 0.33 | 0.64 (-1.7, 3.0) | 0.60 | 0.0 (-2.3, 2.4) | 0.98 | -1.5 (-3.7, 0.7) | 0.19 | Ref. | 0.10 |
| adjusted | 0.2 (-2.0, 2.5) | 0.83 | 0.3 (-2.0, 2.5) | 0.82 | -0.6 (-2.8, 1.7) | 0.62 | -0.9 (-3.0, 1.1) | 0.40 | Ref. | 0.56 |
|  | | | | | | | | | |  |
| **Dietary Pattern 3** | n=188 |  | n=249 |  | n=287 |  | n=283 |  | n=338 |  |
| Mean eGFR (SD) ml/min/1.73m^2^ | 79.1 (14.0) |  | 77.9 (14.0) |  | 80.3 (14.4) |  | 81.9 (12.8) |  | 82.4 (13.5) |  |
| Mean difference in eGFR (ml/min/1.73m^2^) from reference category (95% CI): | | | | | | | | | |  |
| unadjusted | -3.3 (-5.7, -0.8) | 0.009 | -4.4 (-6.7, -2.2) | <0.001 | -2.1 (-4.3, 0.1) | 0.06 | -0.5 (-2.7, 1.7) | 0.65 | Ref. | <0.001 |
| adjusted | -2.3 (-4.7, 0.1) | 0.07 | -3.2 (-5.4, -1.0) | 0.005 | -1.5 (-3.6, 0.6) | 0.16 | -0.0 (-2.1, 2.0) | 0.98 | Ref. | 0.002 |

Abbreviations: eGFR: estimated glomerular filtration rate (CKD EPI SCr & SCys); CI: Confidence interval. Mean eGFR values are unadjusted. Adjusted models included age, sex, diabetes status, systolic blood pressure, smoking status, alcohol consumption, waist circumference, high density lipoprotein, low density lipoprotein, lipid modifying agent use, antihypertensive and diabetes drug use, multiple-deprivation score, and education level.

**Supplementary Table 2.** Mean renal function (unadjusted) and mean difference in renal function (unadjusted and adjusted) by quintiles of dietary pattern adherence for age > 65 years.

|  | **Least**  **adherent** | **p** | **Low**  **adherence** | **p** | **Middle adherence** | **p** | **High adherence** | **p** | **Highest adherence** | **P for trend** |
| --- | --- | --- | --- | --- | --- | --- | --- | --- | --- | --- |
| **Dietary Pattern 1** | n=240 |  | n=267 |  | n=241 |  | n=245 |  | n=252 |  |
| Mean eGFR (SD) ml/min/1.73m^2^ | 64.1 (15.4) |  | 64.3 (16.2) |  | 66.0 (14.8) |  | 66.8 (5.4) |  | 68.4 (14.3) |  |
| Mean difference in eGFR (ml/min/1.73m^2^) from reference category (95% CI): | | | | | | | | | |  |
| unadjusted | -4.3 (-7.0, -1.6) | 0.002 | -4.1 (-6.7, -1.5) | 0.002 | -2.4 (-5.1, 0.3) | 0.08 | -1.6 (-4.3, 1.1) | 0.24 | Ref. | <0.001 |
| adjusted | -2.1 (-4.6, 0.33) | 0.09 | -1.9 (-4.3, 0.4) | 0.11 | -1.7 (-4.0, 0.7) | 0.16 | -1.3 (-3.7, 1.0) | 0.27 | Ref. | 0.09 |
|  | | | | | | | | | |  |
| **Dietary Pattern 2** | n=252 |  | n=273 |  | n=268 |  | n=222 |  | n=230 |  |
| Mean eGFR (SD) ml/min/1.73m^2^ | 70.4 (15.3) |  | 65.2 (15.1) |  | 64.4 (15.1) |  | 64.1 (15.1) |  | 65.9 (15.3) |  |
| Mean difference in eGFR (ml/min/1.73m^2^) from reference category (95% CI): | | | | | | | | | |  |
| unadjusted | 5.0 (2.3, 7.7) | <0.001 | -0.1 (-2.8, 2.6) | 0.94 | -0.9 (-3.6, 1.8) | 0.50 | -1.3 (-4.1, 1.5) | 0.37 | Ref. | <0.001 |
| adjusted | 3.5 (1.0, 6.0) | 0.005 | -0.7 (-3.1, 1.6) | 0.53 | -0.4 (-2.8, 1.9) | 0.71 | -1.9 (-4.3, 0.5) | 0.12 | Ref. | 0.006 |
|  | | | | | | | | | |  |
| **Dietary Pattern 3** | n=326 |  | n=277 |  | n=234 |  | n=228 |  | n=180 |  |
| Mean eGFR (SD) ml/min/1.73m^2^ | 64.8 (14.6) |  | 63.6 (15.7) |  | 66.0 (15.6) |  | 67.1 (15.1) |  | 70.0 (15.1) |  |
| Mean difference in eGFR (ml/min/1.73m^2^) from reference category (95% CI): | | | | | | | | | |  |
| unadjusted | -5.2 (-7.9, -2.4) | <0.001 | -6.4 (-9.2, -3.5) | <0.001 | -3.9 (-6.9, -1.0) | 0.009 | -2.9 (-5.9, 0.1) | 0.06 | Ref. | <0.001 |
| adjusted | -1.8 (-4.5, 0.8) | 0.17 | -3.6 (-6.2, -1.0) | 0.006 | -2.1 (-4.7, 0.5) | 0.12 | -1.3 (-3.9, 1.3) | 0.34 | Ref. | 0.06 |

Abbreviations: eGFR: estimated glomerular filtration rate (CKD EPI SCr & SCys); CI: Confidence interval. Mean eGFR values are unadjusted. Adjusted models included age, sex, diabetes status, systolic blood pressure, smoking status, alcohol consumption, waist circumference, high density lipoprotein, low density lipoprotein, lipid modifying agent use, antihypertensive and diabetes drug use, multiple-deprivation score, and education level.

**Supplementary Table 3.** Adjusted mean difference in renal function (adjusted) by quintiles of dietary pattern adherence for all study participants and by 65 < age > 65 years.

|  | **Least**  **adherent** | **p** | **Low**  **adherence** | **p** | **Middle adherence** | **p** | **High adherence** | **p** | **Highest adherence – mean eGFR** | **P for trend** |
| --- | --- | --- | --- | --- | --- | --- | --- | --- | --- | --- |
| **Dietary Pattern 1** |  |  |  |  |  |  |  |  |  |  |
| All – adjusted (n = 2590) | -3.4 (-5.0, -1.7) | <0.001 | -2.7 (-4.3, -1.1) | 0.001 | -2.5 (-4.1, -0.9) | 0.002 | -0.9 (-2.5, 0.7) | 0.28 | Ref. | <0.001 |
| Age <65 yrs – adjusted (n = 1345) | -4.6 (-6.8, -2.3) | <0.001 | -3.1 (-5.3, -0.9) | 0.006 | -3.0 (-5.2, -0.9) | 0.005 | -0.3 (-2.4, 1.8) | 0.78 | Ref. | <0.001 |
| Age > 65 yrs adjusted (n = 1245) | -2.1 (-4.6, 0.33) | 0.09 | -1.9 (-4.3, 0.4) | 0.11 | -1.7 (-4.0, 0.7) | 0.16 | -1.3 (-3.7, 1.0) | 0.27 | Ref. | 0.09 |
|  | | | | | | | | | |  |
| **Dietary Pattern 2** |  |  |  |  |  |  |  |  |  |  |
| All - adjusted (n = 2590) | 1.9 (0.2, 3.5) | 0.03 | -0.3 (-1.9, 1.3) | 0.71 | -0.7 (-2.3, 0.9) | 0.38 | -1.2 (-2.8, 0.4) | 0.15 | Ref. | 0.02 |
| Age <65 yrs – adjusted (n = 1345) | 0.2 (-2.0, 2.5) | 0.83 | 0.3 (-2.0, 2.5) | 0.82 | -0.6 (-2.8, 1.7) | 0.62 | -0.9 (-3.0, 1.1) | 0.40 | Ref. | 0.56 |
| Age >65 yrs – adjusted (n = 1245) | 3.5 (1.0, 6.0) | 0.005 | -0.7 (-3.1, 1.6) | 0.53 | -0.4 (-2.8, 1.9) | 0.71 | -1.9 (-4.3, 0.5) | 0.12 | Ref. | 0.006 |
|  | | | | | | | | | |  |
| **Dietary Pattern 3** |  |  |  |  |  |  |  |  |  |  |
| All - adjusted (n = 2590) | -1.8 (-3.5, -0.01) | 0.05 | -3.2 (-4.8, -1.5) | <0.001 | -1.5 (-3.1, 0.2) | 0.08 | -0.4 (-2.0, 1.3) | 0.65 | Ref. | 0.001 |
| Age <65 yrs – adjusted (n = 1345) | -2.3 (-4.7, 0.1) | 0.07 | -3.2 (-5.4, -1.0) | 0.005 | -1.5 (-3.6, 0.6) | 0.16 | -0.0 (-2.1, 2.0) | 0.98 | Ref. | 0.002 |
| Age >65 yrs – adjusted (n = 1245) | -1.8 (-4.5, 0.8) | 0.17 | -3.6 (-6.2, -1.0) | 0.006 | -2.1 (-4.7, 0.5) | 0.12 | -1.3 (-3.9, 1.3) | 0.34 | Ref. | 0.06 |

Abbreviations: eGFR: estimated glomerular filtration rate (CKD EPI SCr & SCys); CI: Confidence interval. Adjusted models included age, sex, diabetes status, systolic blood pressure, smoking status, alcohol consumption, waist circumference, high density lipoprotein, low density lipoprotein, lipid modifying agent use, antihypertensive and diabetes drug use, multiple-deprivation score, and education level.
